# If you can’t measure it- you can’t change it – a longitudinal study on improving quality of care in hospitals and health centers in rural Kenya

**DOI:** 10.1186/s12913-018-3052-7

**Published:** 2018-04-05

**Authors:** Michael Marx, Christine Nitschke, Maureen Nafula, Mabel Nangami, Marc Brodowski, Irmgard Marx, Helen Prytherch, Charles Kandie, Irene Omogi, Friederike Paul-Fariborz, Joachim Szecsenyi

**Affiliations:** 1 Institute of Public Health, University of Heidelberg, Heidelberg, Germany; 2 Institute of Public Health, University of Heidelberg, Heidelberg, Germany; 3 Institute of Health Policy, Management and Research (IHPMR), Nairobi, Kenya; 4 Moi University, Eldoret, Kenya; 5 Institute for Applied Quality Improvement & Research in Health Care (AQUA), Göttingen, Germany; 6 Evaplan at the University Hospital, Heidelberg, Germany; 7 Swiss Tropical and Public Health Institute (TPH), Basel, Switzerland; 8 Department of Health Standards, Quality Assurance and Regulations, Ministry of Health, Nairobi, Kenya; 9 Deutsche Gesellschaft für Internationale Zusammenarbeit (GIZ) GmbH, Health Programme, Nairobi, Kenya

**Keywords:** Quality of health care, Quality improvement, Delivery of health care

## Abstract

**Background:**

The Kenyan Ministry of Health- Department of Standards and Regulations sought to operationalize the Kenya Quality Assurance Model for Health. To this end an integrated quality management system based on validated indicators derived from the Kenya Quality Model for Health (KQMH) was developed and adapted to the area of Reproductive and Maternal and Neonatal Health, implemented and analysed.

**Methods:**

An integrated quality management (QM) approach was developed based on European Practice Assessment (EPA) modified to the Kenyan context. It relies on a multi-perspective, multifaceted and repeated indicator based assessment, covering the 6 World Health Organization (WHO) building blocks. The adaptation process made use of a ten step modified RAND/UCLA appropriateness Method. To measure the 303 structure, process, outcome indicators five data collection tools were developed: surveys for patients and staff, a self-assessment, facilitator assessment, a manager interview guide. The assessment process was supported by a specially developed software (VISOTOOL®) that allows detailed feedback to facility staff, benchmarking and facilitates improvement plans. A longitudinal study design was used with 10 facilities (6 hospitals; 4 Health centers) selected out of 36 applications. Data was summarized using means and standard deviations (SDs). Categorical data was presented as frequency counts and percentages.

**Results:**

A baseline assessment (T1) was carried out, a reassessment (T2) after 1.5 years. Results from the first and second assessment after a relatively short period of 1.5 years of improvement activities are striking, in particular in the domain ‘Quality and Safety’ (20.02%; *p* < 0.0001) with the dimensions: use of clinical guidelines (34,18%; *p* < 0.0336); Infection control (23,61%; p < 0.0001). Marked improvements were found in the domains ‘Clinical Care’ (10.08%; *p* = 0.0108), ‘Management’ (13.10%: p < 0.0001), ‘Interface In/out-patients’ (13.87%; *p* = 0.0246), and in total (14.64%; p < 0.0001). Exemplarily drilling down the domain ‘clinical care’ significant improvements were observed in the dimensions ‘Antenatal care’ (26.84%; *p* = 0.0059) and ‘Survivors of gender-based violence’ (11.20%; *p* = 0.0092). The least marked changes or even a -not significant- decline of some was found in the dimensions ‘delivery’ and ‘postnatal care’.

**Conclusions:**

This comprehensive quality improvement approach breathes life into the process of collecting data for indicators and creates ownership among users and providers of health services. It offers a reflection on the relevance of evidence-based quality improvement for health system strengthening and has the potential to lay a solid ground for further certification and accreditation.

## Background

In the drive to achieve universal health coverage (UHC) the importance of quality of care has been accentuated by the 2030 Sustainable Development Agenda. Challenges in service delivery, efficiency and resource utilization in the health sector remain regardless of recent progress.

In recent years, quality of health care has ascended high on the international health agenda especially in the context of Health System Strengthening (HSS) and UHC. Mortality and morbidity rates haven’t declined accordingly [1] although health care utilization rates increased in some low and middle income countries (LMIC). [2, 3] This discrepancy might be expounded by the low quality of care provided in both the public and private sector [4].

In Kenya, but also on a global scale, large and often unexplained differences in quality assessments can be observed between hospitals, facilities and providers. This raises the question of whether these are true differences or the result of weak measurement methods or quality auditors’ biases [5, 6]. Given the multitude of QI tools and approaches in use, it is one of today’s major challenges to improve their compatibility with specific health systems and to take existing instruments, procedures, and data from respective health information systems into account. There is an increasing demand – not only in Kenya – to implement evidence-based QI across health systems to ensure that QI approaches, standards and indicators adhere to scientific standards [5].

In Kenya, as in many other LMIC, remarkable endeavours have been made by the government, development partners, faith based organizations and the private sector to improve service delivery, efficiency and resource utilization. However, service performance and health indicators stay behind in the Kenyan health sector.

Besides deficient infrastructure and shortages of equipment, drugs and staff problems of quality of care are prevailing. These are particularly distinctive in the areas of maternal and neonatal care, family planning and in the provision of services for the survivors of sexual and gender-based violence [7]. Hence the maternal mortality rate remains intolerably high at 362 per 100,000 live births [8]. Whereas health facility data indicated that 95.7% of pregnant women in Kenya attended at least one antenatal care (ANC) visit in 2014, the minimum of four ANC visits, as recommended by World Health Organization (WHO), was only accessed by 57.6% according to survey data. More than half of pregnant women (61%) delivered at a health facility in 2014 [9]. But even these facility-based deliveries are often performed under inadequate professional surveillance [10]. The availability and use of essential guidelines at facility level is not warranted [11]. In 2014, contraceptive prevalence was still low, with not much more than half of married women in Kenya (58%) using any method and often contraceptives are out of stock [7]. Women’s increased vulnerability to HIV infection has been particularly connected to gender based violence as a special act of defiance, the seriousness of which has been repeatedly shown in the Kenyan context [12–14].

In 2001 the Kenya Quality Model (KQM) was launched by the Ministry of Health (MoH) [15]. KQM defined quality management as a process to better comply with standards and guidelines, to improve structures, processes and results in health care by Quality management (QM) tools and to meet patient needs. However, KQM was not implemented in a participatory way and remained a frozen tool [16]. KQM was therefore revised, extended and renamed into Kenya Quality Model for Health (KQMH). KQMH is supposed to serve as the national framework to unify existing approaches to improve quality of care at all facilities of the health system. Although KQMH has been further developed into a comprehensive conceptual framework for QM challenges remain to operationalize KQMH. In response to this implementation gap, and as part of its support for the Kenyan health sector, the Deutsche Gesellschaft für Internationale Zusammenarbeit (GIZ) sought to support the Kenyan Ministry of Health’s Department of Standards and Regulatory Services (DSRS) to establish a practical modality to operationalize the KQMH and make it the point of reference for all facilities working to improve the quality of their services. An integrative methodology was needed to reduce fragmentation, while an evidence-based approach was sought to strengthen the knowledge about how improving the quality of care can strengthen health systems.

A consortium including evaplan GmbH at the University of Heidelberg, the Institute for Applied Quality Improvement & Research in Health Care in Germany (aQua), and the Institute of Health Policy, Management and Research (IHPMR) in Nairobi was contracted for the development and implementation of an Integrated Quality Management System (IQMS). A first assessment of quality improvement activities that included a stakeholder mapping revealed a rather piecemeal approach to the topic of quality improvement in Kenya. Moreover, though traditional tools like supervision, the use of a Health Management Information System (HMIS) and continuous professional training were widely applied, the efforts did not produce expected results in terms of improved health outcomes.

Supervision was carried out erratically and the full potential of the approach was not exhausted. Modern quality tools like self-assessment were not well-known and little used. The completion of reporting forms was often undertaken late, the data itself was of questionable quality and the extent to which the data was used to inform health facility and sector planning limited [17].

This paper describes both the development and implementation of the IQMS and demonstrates how such an integrated quality management approach can serve as a powerful tool for decision making in poor resource settings and hence significantly improve the quality of care.

## Methods

The aQua-Institute has developed a systemic, comprehensive and evidence-based Quality Management tool for the German health system. This integrated Quality Management approach has been formalized into the European Practice Assessment (EPA) and since 2013 is being implemented in more than 3000 health facilities in Germany, in the Netherlands, Belgium, Romania, Austria, Switzerland and Slovenia. It is a multiperspective, multifaceted indicator based approach that covers five domains (infrastructure, people, information, finance, and quality & safety) covering most of the six WHO health system building blocks. These domains can be modified according to the needs of the country and its health facilities.

A specially developed software (VISOTOOL®) visualizes the results in an easily understandable way to stimulate discussion with facility staff and facilitate the development of highly tailored improvement plans. Furthermore the software allows facilities to benchmark their results against the average result of all participating facilities [18, 19].

To operationalize the Kenya Quality Assurance Model for Health, EPA was adapted to leverage its integrative and evidence-based indicator-based approach in collaboration with the Ministry of Health including the Department of Standards and Regulations, the Department of Clinical Services, the Division of HMIS, the Division of Reproductive Health, the Division of Child Health and the Unit of Monitoring & Evaluation.

**Fig. 1:**
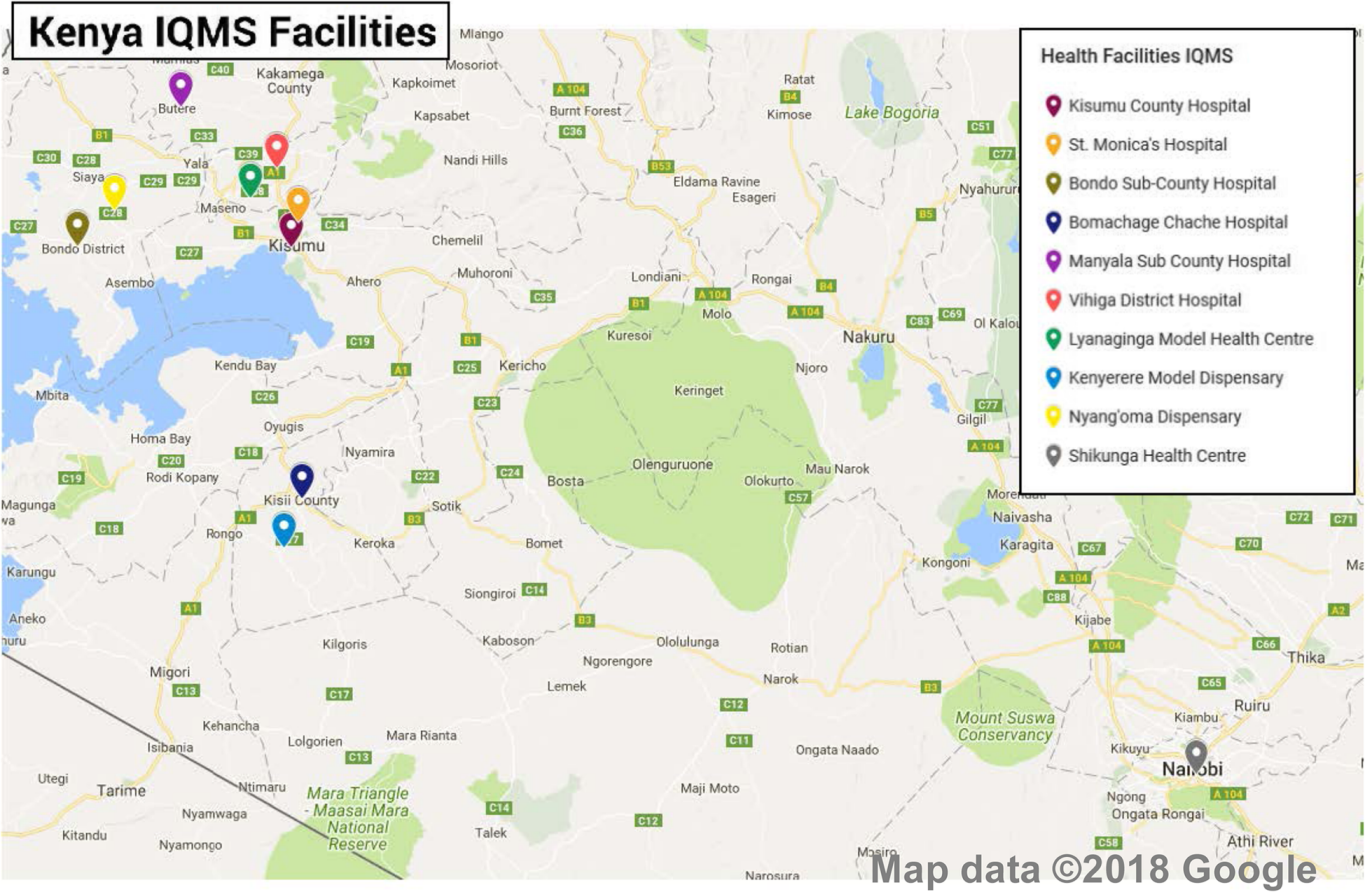
Map of Kenya with distribution of facilities

**Fig. 2:**
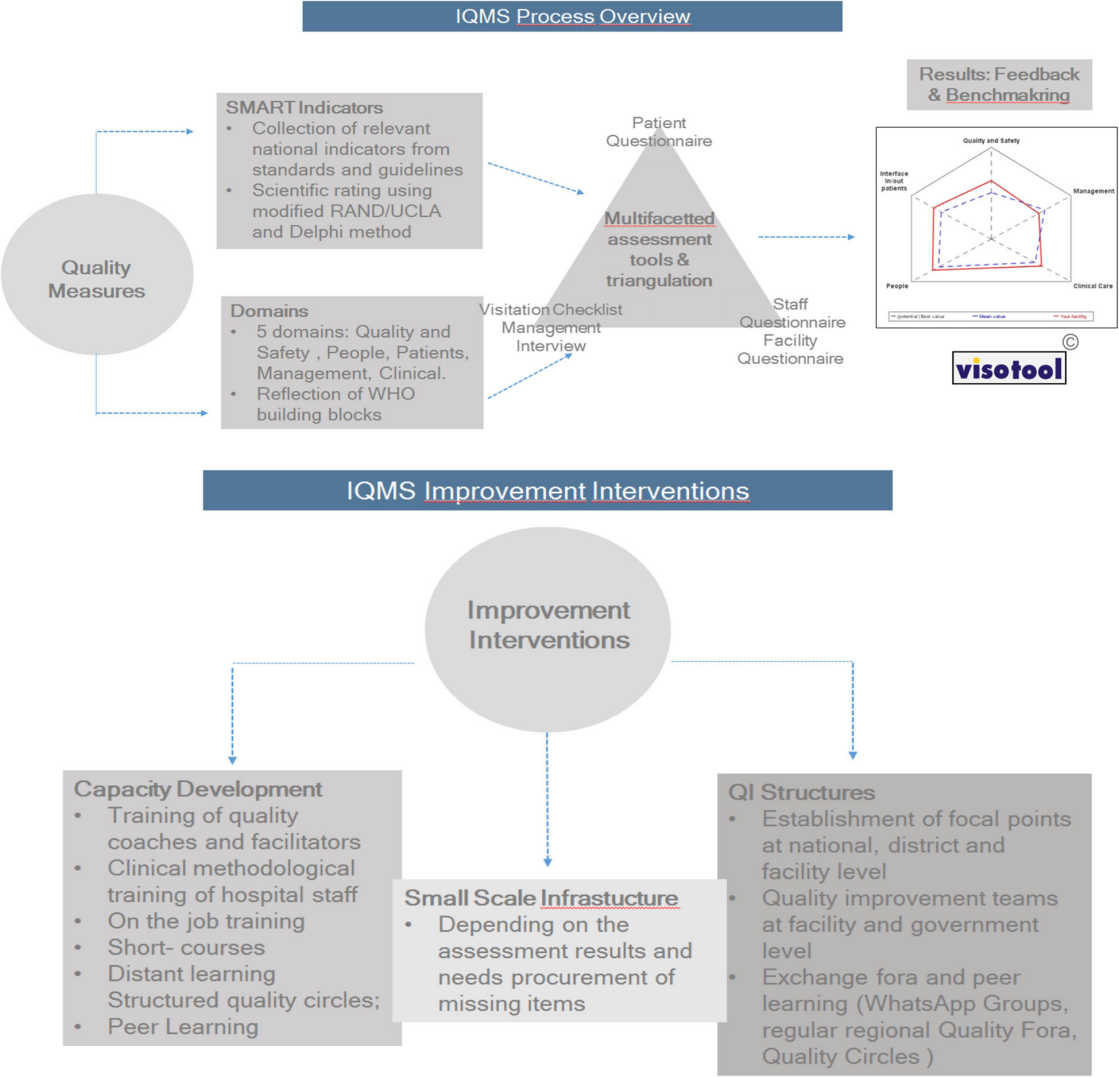
Diagram outlining IQMS process

The adaptation process made use of a ten step modified RAND/UCLA appropriateness method. This systematic method to validate indicators is described in detail by Prytherch et al. [5]. The steps included a scoping workshop, definition of five critical domains of quality in the Kenyan context, and a review of more than 50 policy and planning documents, standards, management and clinical guidelines, grey and scientific literature to identify indicators in use in the Kenyan health system. An expert panel adapted and validated the five proposed domains, and assessed the identified, candidate indicators according to the Specific, Measurable, Achievable, Relevant and Time-bound (SMART) criteria, before rating them on their validity and feasibility using a modified Delphi method. The resulting 303 structure, process and outcome indicators, clustered across the five domains (Clinical Care, People, Management, Interface In/out-patients, and Quality and Safety), were broken-down into 29 dimensions. For the domain Clinical Care illustrative dimensions include antenatal care, delivery, postnatal care, family planning, survivors of gender-based violence; for the domain People they include patient satisfaction, staff satisfaction, staff general, staff appraisal, staff support; for the domain Management they include leadership and governance, financial, maintenance, supplies, drugs, data, equipment, amenities, transport, waiting times; for the domain Interface In/out-patients they include community, general, referral; for the domain Quality and Safety they include general, guidelines etc., critical incident reporting, emergency management, infection control, laboratory. Finally, a set of five data collection tools based upon the final register of indicators were developed. Following the principle of triangulation of methods these tools included surveys for patients and staff, a self-assessment, facilitator assessment and a manager interview guide. The data collection tools where then incorporated into the specially developed software (VISOTOOL®). The use of quality indicators is described in detail in Goetz et al. [20] and Herrler et al. [21] (Figs. 1 and 2).

### Design and sampling

A longitudinal study design was used. Ten facilities were selected out of 36 applications representing a variety of Kenyan facilities of all levels of care and from both rural and urban settings (*n* = 10; six hospitals (including four district hospitals and two county hospitals): Kisumu County Hospital, St. Monica’s Hospital, Bondo Sub County Hospital, Bomachage Chache Hospital, Manyala Sub County Hospital and Vihiga County Hospital; four health centers: Lynaginga Model Health Centre, Kenyerere Model Dispensary, Nyan’goma Dispensary, Shikunga Health Centre). The selection was purposeful but based on criteria like a specified minimum level of infrastructure and medical equipment for reproductive health-care provision, service provision for survivors of gender based violence, previous experience in the field of QM and motivation to invest in quality assurance in the long run. These selection criteria were to ensure the comparability between health facilities and reduce structural variables that might affect the generalisation of findings. The selected facilities were first visited in 2013 (T1) and re-visited between September 2015 and February 2016 (T2). Based on the identified gaps at T1, target-oriented improvement plans were developed with the facilities and in between the measurements several interventions were carried out in the facilities under continuous supervision.

The domain People with 85 indicators has been excluded from this analysis due to being personal data and will be published separately. All calculated values are based on the percentage achievements of the remaining 218 indicators, 24 dimensions and four domains (Clinical Care, Management, Interface In/out-patients, and Quality and Safety) at T1 and T2 on scale of 0 to 100 for each facility. All indicators, dimension and domain values of T1 and T2 for each facility have been exported from the software VISOTOOL®. Data was summarized using means and standard deviations (SDs). Categorical data was presented as frequency counts and percentages. Since the double-time data is not complete for all indicators at every facility, the calculations of the percentage changes had to be based only on those indicators with available values at both T1 and T2 at each facility. A z- testing was first considered, but due to the low data variance a t- test was felt more appropriate. *p*-values have been calculated in MicrosoftExcel2010 applying the one sample t-test on the mean change (difference: T2 minus T1 value) and the standard deviation. Since positive and negative changes are possible, a double-tailed event has been chosen and the expected value has been defined as a change 0, following the null hypothesis that the IQMS quality improvement has no significant impact on the T2 value for each indicator with both values. A significance level of α = 0.05 for a confidence interval of 95% has been chosen and a change (as improvement if positive or deterioration if negative) is significant if p < α, leading to a refusal of the null hypothesis.

### Assessment process

The data collection tools were field tested in two facilities. Ethical clearance was obtained from the Institutional Research Ethics Committee *(*IREC) at Moi University, Kenya. The confidentiality of the analysis process and the fact that all responses would be depersonalized was emphasized and all participants provided informed consent.

The project was executed in two phases between 2013 and 2016. A baseline assessment was carried out during the first phase (T1) a reassessment (T2) after 1.5 years. All 10 facilities enrolled completed the first (baseline) and the second assessment.

Each assessment was implemented in two rounds making use of the above-described tools: surveys for patients and staff, a self-assessment, facilitator assessment and a manager interview guide. Experienced research assistants were used to carry out the patient survey orally in English and local languages. At least 100 responses per facility were sought from patients attending Antenatal Care, Post Natal, Family Planning and Maternity services. These surveys were complemented with the information received from the facility managers via their self-assessment. The data from these surveys was entered remotely into the VISOTOOL® software by a research assistant for analysis. A trained facilitator oversaw the facility assessment process, and the training of national “quality facilitators”. Self-assessment and patient and staff surveys were followed by a visitation through a trained facilitator, following a checklist and conducting a management interview, data was immediately entered into VISTOOL® and analysed on site.

Using VISOTOOL® the assessment was followed by an immediate and comprehensive feedback to the health facility staff. This enabled facilities to identify and focus on priority areas. Concrete and highly tailored plans of action were elaborated, preferably making use of locally available resources, including making use of existing quality improvement approaches such as KAIZEN-5S, coaching and quality circles (Tables 1 and 2).

**Table 1:** Indicators from the Domain Clinical Care, Dimension Delivery & Newborn Care with source [5]

Percentage of macerated still births as proportion of total deliveries at facility in the last 12 months	International Indicator WHO
Percentage of pregnant women admitted into maternity with unknown HIV status that are counselled and tested for HIV during labour or after delivery during last month	PMTCT Guideline p.90
Percentage of HIV positive mothers admitted in maternity taking or reported to have taken the mother doses of preventive ARV prophylaxis during last month	PMTCT Guideline p.90
Percentage of infants born in facility receiving infant preventive ARV prophylaxis in maternity clinic during last month	PMTCT Guideline p.90, Health Sector 2nd Ed. Indicators, SOP Manual (HIS), 2011 p.58
Percentage of deliveries conducted by certified staff in the last 12 months	Health Sector Indicators and Standard Procedures - Popular Version p.4, Health Sector 2nd Ed Indicators and SOP Manual (HIS), 2011 p.5,
Percentage of Newborns with Low Birth Weights (LBW) –(less than 2500 g)	Health Sector Indicators and Standard Procedures - Popular Version p.4, Health Sector 2nd Ed Indicators and SOP Manual (HIS), 2011 p.26
Percentage of maternal death reported at facility level in the last 12 months (calendar)year	Health Sector Indicators and Standard Procedures - Popular Version p.6, Kenya Quality Assurance Model for Health Level 3 and 4 Check list, 2009 p.28, Hospital reforms Supervision and Monitoring Tool 2010-2011 p.8, DRH, M&E Framework, 2011-2012 p.21
Percentage of perinatal deaths at the facility in the last 12 months (calendar year)	Kenya Quality Assurance Model for Health Level 3 and 4 Check list, 2009 p.28
Percentage of fresh still births as proportion of total deliveries at facility in the last 12 months	International Indicator WHO
Percentage of births where correctly filled out partographs were used in the last month	Kenya National Reproductive Health Output Based Quality Improvement Accreditation and Assessment Tool, Page 14
The Facility has basic delivery equipment as per essential commodity list, the equipment is functional and maintained (scissors or blade, suction apparatus, disinfectant for cleaning perineum)	Kenya Service Provision Assessment (KSPA) 2010 p.136; Norms and Standards
Percentage of Perinatal Deaths Audited	New Indicator, added by the panel at the first workshop

**Table 2:** Measurement of indicators of Table 1 across the different assessment tools [5]

Staff survey		
	There is good collaboration between my facility and traditional birth atendants	Likert scale 1 (strongly agree)-5 (strongly disagree)
Patient survey	Questions (asked of maternity patients only)	
	Were you ensured of privacy at the delivery?	Y/N
	Did you get a hot drink after the delivery?	Y/N
	Did you get anything to eat after the delivery?	Y/N
	Did you receive sanitary pads after the delivery?	Y/N
	Were you given warm bathing water after the delivery?	Y/N
Self-assessment		
	Total number of macerated still births at the facility in the last 12 months	Provide number from maternity register
	Total number of fresh still births at the facility in the last 12 months	Provide number from maternity register
	Total number of deliveries in the facility in the last 12 months	Provide number from maternity register
	Number of maternal deaths in the facility in the last 12 months	Provide number from maternity register
	Total number of perinatal deaths	Provide number from maternity register
	Total number of live births at the facility during the last 12 months	Provide number from maternity register
Facilitator checklist		Instruction
	Total number of correctly filled out partographs in the last month	Look at the documentation of 10 randomly selected deliveries in the last month and enter number of times this was the case
	Does the facility practice kangaroo mother care	Y/N
	If yes, can staff members give a demonstration and explain when and how it should be used?	Y/N
	*in the equipment dimension*	
	The following basic equipment is available and functional: Weighing scale for newborns, scissors/blade, suction apparatus, disinfectant for cleaning perineum, drip stand, torches/portable lights	Y/N in each case. Yes only to be ticked if equipment is both available and functional on day of assessment
	*in the amenities dimension*	
	Are the following basic amenities for service provision of maternity unit for level 2 and 3 available according to norms and standards: three examination coaches, three screens, two delivery beds, 10 delivery kits, one resuscitation tray, oxygen, incubator, maternity beds, MWV kids, five stiching trays, CS kits, etc	Y/N in each case
	Does the labour ward provide privacy for clients?	Y/N in each case
	*in the infection control dimension*	
	Does the facility have a functional placental pit; is it lockable, is it concretelined with depth greater than 1 m, is it inside the facility compound secured from unauthorised access?	Y/N in each case
	*in the drugs dimension*	
	Are the following available on day of assessment: antibiotics for newborn sepsis according to guidelines; ARVs or PMTCT according to guidelines; oxytocic according to guidelines, dextrose 5%; normal saline; ringer lactate; IV infusion set etc	Y/N in each case
	*also aspects covered in the supplies, referral and community interface dimensions*	
Manager interview		
	Are the standard clinical guidelines available for active management of 3rd stage of labour?	Y/N
	Is the implementation of this guideline in the daily routine work discussed with members of the clinical team?	Y/N
	*in the community dimension*	
	Do health promotion activities covering the importance of delivering at a facility take place at least quarterly?	Y/N
	*also aspects covered in the referral and critical incident reporting dimensions*	

Between T1 and T2 the facilities used the given analysis and feedback of assessment T1 results for decision-making on what intervention should be given priority and be implemented. Each of the 10 facilities then conducted a number of one to five improvement interventions based on the gaps identified and accompanied by facility-driven tutoring and coaching targeting five main topics: neonatal mortality, the completeness of partographs, waiting times, IPC as well as shortages of staffing and transportation in remote areas.

Facilities were grouped according to whether or not a concrete improvement intervention was conducted. Only those improvement intervention topics with group sizes of at least two participating and two not-participating facilities were considered as eligible for a comparison of their T1 and T2 results, in respect to those IQMS indicators matching their mentioned inducements and intervention contents (Table 3).

**Table 3:** Summarized inducements, number of relevant and final IQMS indicators and intervention contents

Improvement intervention topic	Inducement	N° relevant indicators (with performed analysis)	Intervention contents
Neonatal mortality	High neonatal mortality rates	72 (27)	Root cause analysis, auditing of all perinatal deaths, a creation of a separate newborn unit, minor renovations and improving of IPC practices
Completeness of partograph	Low percentages of sampled partographs correctly filled	11 (4)	Conduction of CME (Continuing Medical Education) for staff and the institution of monitoring
Waiting times	Longer than promised to clients waiting times	12 (11)	Introduction of a customer desk, the sensitization of all departments and units and an overall introduction of customer flow systems
IPC	IPC not meeting the required standards	24 (20)	Training and implementation of 5S, training on root cause analysis, improvement in IPC practices and a carrying out of regular assessments
Shortages of staffing and transportation in remote areas	Transferring out of staff and a poor public transport system	15 (12)	Improvement on the referral system, establishment of a good communication with coordination of ambulances, attendance to all emergency cases within 45 min and the possibility of referral

## Results

The characteristics of the study population are listed in Table 4.

**Table 4:** Characteristics of the study population

Level of service	Total number	Region		Ownership		Total number of beds (mean (range))	Catchment population (mean (range))
		Rural	Urban	Public	Faith based		
Health Center	4	3	1	3	1	20.5 (12-37)	12797.5 (6927-17607)
Hospital	6	2	4	5	1	98.5 (30-180)	136507.33 (16759-473649)

Excluding the fifth domain ‘People’, changes in the scores of the four domains and all 24 dimensions for the ten facilities at the two assessments are shown in Table 5.

**Table 5:** Total number of indicators, T1 (first assessment) and T2 (re-assessment) mean scores, percentage change, standard deviation and *p* values

Domains	Dimensions	Number of indicators	T1 (n = 10) ‡	T2 (n = 10) ‡	Average percentage change (difference T2-T1) ‡ of all relying indicators with available values at two times	Standard deviation	P-value
Clinical Care		86	62.66	72.73	10.08	24.12	0.0108
	1 Antenatal Care	13	53.69	80.54	26.84	27.29	0.0059
	2 Delivery	7	85.68	75.47	−10.21	24.41	0.3524
	3 Postnatal Care	6	72.05	67	−5.05	21.42	0.6259
	4 Family Planning	8	65.55	73.60	8.04	19.51	0.2818
	5 Survivors of gender-based violence	11	53.50	64.70	11.20	10.74	0.0092
Management		90	68.41	81.52	13.10	16.72	< 0.0001
	1 Leadership and governance	17	62.75	71.73	8.98	20.31	0.0971
	2 Financial	5	83.02	92.03	9.01	11.07	0.1431
	3 Maintenance	4	40.06	53.31	13.25	24.96	0.3662
	4 Supplies	7	68.63	94.8	26.17	20.36	0.0145
	5 Drugs	15	79.34	92.12	12.78	14.94	0.0051
	6 Data	5	65.86	80.90	14.94	11.17	0.0403
	7 Equipment	3	65.56	66.34	0.79	13.83	0.9303
	8 Amenities	23	65.13	77.92	12.79	13.70	0.0003
	9 Transport	2	77.5	95	17.5	17.68	0.3949
	10 Waiting times	9	72.99	88.86	15.87	19.05	0.0369
Interface In/out-patients		16	58.64	72.51	13.87	22.21	0.0246
	1 Community	2	69.41	95.66	26.26	13.37	0.2201
	2 General	3	84.90	78.25	−6.66	30.81	0.7442
	3 Referral	11	49.52	66.74	17.22	19.04	0.0133
Quality & Safety		67	51.13	71.15	20.02	18.51	< 0.0001
	1 General	1	35.71	21.43	57.14		
	2 Guidelines etc.	4	41.73	75.9	34.18	18.32	0.0336
	3 Critical incident reporting	7	34.92	50.04	15.12	19.44	0.0853
	4 Emergency management	4	43.73	54.75	11.03	14.95	0.2367
	5 Infection control	24	51.75	75.35	23.61	19.41	< 0.0001
	6 Laboratory	27	58.47	75.76	17.30	17.69	< 0.0001
Total		218	61.30	75.93	14.64	19.57	< 0.0001

Significant improvements were found in all four domains with higher scores measured in the domains ‘Clinical Care’ (10.08%; *p* = 0.0108), ‘Management’ (13.10%; *p* < 0.0001), ‘Interface In/out-patients’ (13.87%; *p* = 0.0246), ‘Quality and Safety’ (20.02%; p < 0.0001) and in total (14.64%; p < 0.0001).

In the domain ‘clinical care’ significant improvements were observed in the dimensions ‘Antenatal care’ (26.84%; *p* = 0.0059) and ‘Survivors of gender-based violence’ (11.20%; *p* = 0.0092). The least marked changes or even a -not significant- decline of some was found in the dimensions ‘delivery’ and ‘postnatal care’.

For the domain ‘management’ significant improvements were observed in the dimensions ‘Supplies’ (26.17%; *p* = 0.0145), ‘Drugs’ (12.78%; *p* = 0.0051), ‘Data’ (14.94%; *p* = 0.0403), ‘Amenities’ (12.79%; *p* = 0.0003) and ‘Waiting times’ (15.78%; *p* = 0.0369).

For the domain ‘Interface In/Out-patients’ significant improvements have been observed in the dimension ‘Referral’ (17.22% *p* = 0.0133). The least marked changes or even a -not significant- decline of some was found in the dimension ‘General’.

In the domain ‘quality and safety’ significant improvements were observed in the dimensions ‘Guidelines etc’ (34.18%; *p* = 0.0336), ‘Infection control’ (23.61%; *p* < 0.0001) and ‘Laboratory’ (17.30%; p < 0.0001).

**Fig. 3:**
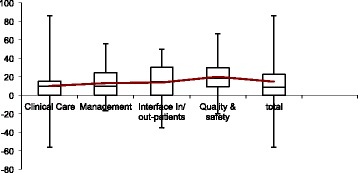
Box-Plot showing the variation of the average indicator changes of each domain.The line connects mean values

The following boxplot (Fig. 3) shows the variation of all indicator changes within each domain. All mean values are within interquartile range and therefore represent the entirety of all values.

As health centres and hospitals are discussed together, a comparison of the average improvements of all 4 health centres with those of all 6 hospitals should prove that structural characteristics are not crucial for the achievement of a significant improvement (Table 6).

**Table 6:** A comparison of the percentage changes with p-values for each domain and in total for the mean of health centres and hospitals

	Clinical Care	Management	Interface In/out-patients	Quality & Safety	total
Health centers	15.27/*p* = 0.0085	9.65/*p* = 0.0002	16.94/*p* = 0.0908	17.08/*p* = 0.0001	13.60/p < 0.0001
Hospitals	9.20/*p* = 0.0578	15.47/p < 0.0001	13.14/*p* < 0.0415	22.01/p < 0.0001	16.01/p < 0.0001

**Fig. 4:**
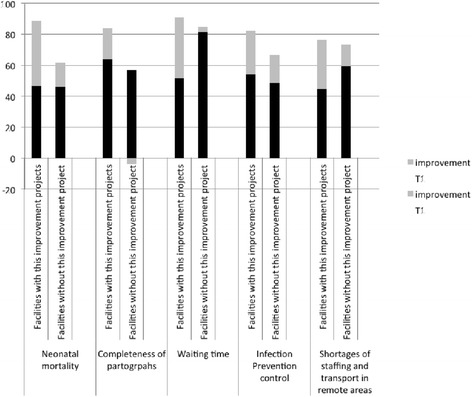
Percentage T1 and improvement (=change (T2-T1) values compared for facilities with (intervention group) and without (non-intervention group) the concrete improvement interventions

The differences between T1 and improvement values (= T2 values), comparing the intervention and the non-intervention groups are shown below (Fig. 4).

Looking at the results of interventions, the analysis showed for example that the improvement interventions conducted to reduce neonatal mortality achieved higher improvement rates (change) (42.33%) than the non-intervention group, where the improvement of the comparable indicators has also been significant (15.57%). Those facilities that implemented concrete activities to improve their IPC, achieved significantly higher improvements (28%) than those facilities that did not. Nevertheless, also for those facilities without concrete IPC interventions marked improvement could be observed (18%).

## Discussion

To improve quality of care various factors and a combination of methods are influential, such as evidence based measurement from different perspectives, extensive feedback to staff and prioritized improvement activities. Intrinsic motivation of staff could be assumed given the selection process to participate. In the mid run, there may be external incentives to embark into a systemic quality improvement process, such as accreditation [20].

Our assumption is that a precise, detailed and participative measurement and gap analysis - as a tool for good decision-making - is a basic requirement for setting the improvement process in motion and leads to effective targeted improvement interventions which are accompanied by facility-driven coaching and tutoring. Analysis on the effectiveness of the precursor of the IQMS Kenya, EPA in Germany and Switzerland, has shown significant improvements in three of four analysed domains and demonstrated the ability of EPA as effective and efficient quality management program [6, 20].

The higher significant achievements of improvement interventions in relation to the comparison group demonstrate the effectiveness of this targeted intervention performed under facility-driven coaching and tutoring. The integral IQMS quality improvement approach demonstrated that those facilities with a combination of measurements including gap analysis, decision-making and the conduction of supervised targeted interventions achieved better improvements than facilities with the same starting conditions, but only the performance of measurement. We can thus assume, that the actual improvement can be attributed to the systemic nature of the approach.

Lower T1 values at the participating facilities – for example in the improvement interventions on waiting time and on shortages of staffing and transport in remote areas - underline the validity of the integral IQMS approach for revealing deficiencies within this area. Despite these deficiencies in comparison to the non-intervention group, all improvement interventions were able to achieve significant changes and even higher T2 values than the non-intervention group, which proves its possibility of not just catching up but overtaking by performing a previous gap analysis and prioritization of concrete interventions [6].

Furthermore, the methodological approach chosen serves different purposes of quality assessment: internal improvement, external accountability, and scientific evidence. Therefor it is paramount to measure structure, process, or outcome of healthcare.

Without looking into all details of the actual improvement process between T1 and T2 - we assume that the precise measurement of quality problems helps sensitize health staff to recognize and accept quality problems, which is also endorsed by other authors [6, 19, 22]. Being a very precise measurement method it proved to be an effective way to improve quality without any additional significant resources. Even the best T1 values in the domain ‘Management’ (68.4%), followed by the domains ‘Clinical Care’ (62.66%) ‘Interface In/out-patients’ (58.64%), and finally ‘Quality & Safety’ (51.13%) still show potential for improvement and therefore demonstrate the necessity for continuous quality improvement, one of the principles of KQMH. On the other hand the high degree of prioritizing certain interventions over others, could also explain that a dimension and the respective indicators reach lower level of improvement in comparison to others.

The approach has the power to integrate different, pre-existing and possibly competing quality improvement (QI) initiatives and to reduce the risk of indicators being reinvented. With the exception of the 44 international indicators that were retained through the review and rating process, 234 of the 303 indicators used had previously existed in the Kenyan health system. In addition to exploring clinical areas the approach offers the possibility to illuminate health system bottlenecks like drug distribution and facility accounting issues. The specially developed software (VISOTOOL®) generates real time results for immediate feedback to the facility team as an integral part of the facility visit process. Precise measurement as well as detailed display of results empower the health facility teams to better analyse underlying problems, set their quality objectives and ensure the optimal use of existing resources according to the Pareto principle. Facilities can also track their progress with the software by comparing results after each assessment. Furthermore the software allows benchmarking. Health facilities can compare their results against the average results of other facilities taking part in the assessments.

Our experience showed that this indicator based approach can be adapted to and used in different contexts and health systems.

Nevertheless, the study has limitations: T2 results might not be a pure reflection of the IQMS quality improvement process, but also be influenced by structural differences regarding staff qualification, availabilities, resources and attitude at the facilities. As to the driving factors for improvement despite structural similarities among the selected health facilities an attribution gap may exist and confounders, e.g. interferences by other health system strengthening activities, could not be excluded. Moreover it can not be clearly defined which factors and improvement activities are producing better results. This is subject of a separate analysis being in process.

## Conclusion

There is a need for validated methods to measure quality of care in LMICs. In accordance with existing literature our results demonstrate that implementing a quality management system based on a systematic performance monitoring of health facilities which includes a continuous improvement process, not only breathes life into the process of collecting data for indicators, but also creates motivation for change and ownership among users and providers of health services and can serve as a powerful tool to improve health outcomes in LMIC.

As such it offers a reflection on the relevance of evidence-based quality improvement for health system strengthening and has the potential to lay a solid ground for further certification and accreditation.

## Abbreviations

ANC: Antenatal care
DSRS: Department of Standards and Regulatory Services)
EPA: European Practice Assessment
GIZ: Deutsche Gesellschaft für Internationale Zusammenarbeit
HIV: Human immunodeficiency virus
HMIS: Health management information system
HSS: Health system strengthening
IHPMR: Institute of Health Policy, Management and Research
IQMS: Integrated quality management system
IREC: Institutional Research Ethics Committee at Moi University, Kenya
KQM: Kenya quality model
KQMH: Kenya quality model for health
LMIC: Low and middle-income countries
MoH: Ministry of health
QI: Quality improvement
QM: Quality management
SD: Standard deviation
SMART: Specific measurable accepted realistic time bound
T1: Time of measurement: first assessment round in 2013
T2: Time of measurement: re-assessment in 2015/2016
UHC: Universal health coverage
WHO: World Health Organization
